# Tongue carcinoma infrequently harbor common actionable genetic alterations

**DOI:** 10.1186/1471-2407-14-679

**Published:** 2014-09-19

**Authors:** Daniel SW Tan, Weining Wang, Hui Sun Leong, Pui Hoon Sew, Dawn P Lau, Fui Teen Chong, Sai Sakktee Krisna, Tony KH Lim, N Gopalakrishna Iyer

**Affiliations:** Cancer Therapeutics Research Laboratory, National Cancer Centre Singapore, 11 Hospital Drive, Singapore, 169610 Singapore; Department of Medical Oncology, National Cancer Centre, 11 Hospital Drive, Singapore, 169610 Singapore; Department of Surgical Oncology, National Cancer Centre, 11 Hospital Drive, Singapore, 169610 Singapore; Department of Pathology, Singapore General Hospital, Outram Road, Singapore, 169610 Singapore

**Keywords:** Druggable, Therapeutic, Squamous cell carcinoma, Sequenom, Oral cancers, HNSCC

## Abstract

**Background:**

Oral tongue squamous cell carcinomas (TSCC) are a unique subset of head and neck cancers with a distinct demographic profile, where up to half of the cases are never smokers. A small proportion of patients with OSCC are known to respond to EGFR TKI. We used a high-sensitivity mass spectrometry-based mutation profiling platform to determine the EGFR mutation status, as well as other actionable alterations in a series of Asian TSCC.

**Methods:**

66 TSCC patients treated between 1998-2009 with complete clinico-pathologic data were included in this study. Somatic mutation profiling was performed using Sequenom LungCarta v1.0, and correlated with clinical parameters.

**Results:**

Mutations were identified in 20/66(30.3%) of samples and involved TP53, STK11, MET, PIK3CA, BRAF and NRF2. No activating EGFR mutations or KRAS mutations were discovered in our series, where just over a third were never smokers. The most common mutations were in p53 (10.6%; n = 7) and MET (10.6%, n = 11) followed by STK11 (9.1%, n = 6) and PIK3CA (4.5%, n = 3). BRAF and NRF2 mutations, which are novel in TSCC, were demonstrated in one sample each. There was no significant correlation between overall mutation status and smoking history (p = 0.967) or age (p = 0.360). Positive MET alteration was associated with poorer loco-regional recurrence free survival (LRFS) of 11 months [vs 90 months in MET-negative group (p = 0.008)]. None of the other mutations were significantly correlated with LRFS or overall survival. Four of these tumors were propagated as immortalized cell lines and demonstrated the same mutations as the original tumor.

**Conclusions:**

Using the Sequenom multiplexed LungCarta panel, we identified mutations in 6 genes, TP53, STK11, MET, PIK3CA, BRAF and NRF2, with the notable absence of EGFR and HER2 mutations in our series of Asian OSCC. Primary cell line models recapitulated the mutation profiles of the original primary tumours and provide an invaluable resource for experimental cancer therapeutics.

## Background

Oral squamous cell carcinoma (OSCC) is a significant world-wide public health threat accounting for approximately 270,000 cases with 145,000 deaths annually [1, 2]. The highest prevalence is seen in developing countries and five-year survival rates remain less than 50% [1–3]. The majority originate from the anterior tongue, and less commonly from the buccal cavity, alveolus, floor of mouth, retromolar trigone and hard palate. Several reports have suggested an increase in incidence of OSCC over recent years, afflicting not only those of lower socioeconomic status and developing countries, but also in developed countries such as the US and UK [4–7]. Moreover, a proportion of cases occur in younger patients who are never smokers, with no relation to betel nut chewing or smoking, common risk factors for oral cancers [8, 9]. There is also evidence to suggest that the various subsites within the oral cavity exhibit significant differences in clinical behavior that are not attributable to the pathogenesis alone [10, 11]. This has prompted several investigators to focus studies on specific subsites, including large scale next-generation sequencing efforts initiated by the International Cancer Genome Consortium (ICGC) [12].

The rapidly expanding repertoire of targeted therapeutics against key somatic alterations has led to increased endeavors towards pathway-driven approaches to treating cancer. For example, head and neck and lung cancers are well known to have activated EGFR pathway, and this has led to focused development of drugs that either directly inhibit the EGFR receptor such as monoclonal antibodies like cetuximab, block the tyrosine kinase activity (small molecules including gefitinib, erlotinib, afatanib etc) or molecules that block the downstream signal transduction cascade (targeting Phosphoinositide 3-kinase (PI3K), mammalian target of rapamycin (mTOR) etc) [13]. Specific activating EGFR mutations act as a predictive marker for tyrosine kinase inhibitors such as gefitinib and erlotinib in NSCLC, and have conferred significant improvement in overall survival [14]. Clinical activity of EGFR tyrosine kinase inhibitors (TKI) has also been examined in head and neck cancer, where responses have been seen in up to 15% but there is no correlation between response and EGFR mutations [15–24]. Few studies have looked for EGFR mutations and other “actionable mutations” in OSCC, and most to date have been conducted in small heterogeneous HNSCC patient cohorts. Studies focused on oral or tongue squamous cell carcinoma show possible population differences in prevalence for EGFR mutation rates, suggesting ethnic differences may exist [24–37]. Similarly, large scale sequencing efforts in HNSCC by several collaborative groups have focused mainly in Caucasian populations, revealing common mutations in genes such as p53, p16, Notch, FAT1, H-Ras and Caspase8 [24, 26, 27, 38]. Recently Zanaruddin et al. reported the use of Sequenom Oncocarta to profile 112 oral SCC samples in Asian patients [39]. While potentially actionable mutations such as PIK3CA and HRAS were reported in that study, tongue cancers only comprised 30% of patients.

Due to the potential similarities among aerodigestive tract cancers, we adopted the Sequenom LungCarta panel to comprehensively evaluate a set of 66 Asian tongue cancers for EGFR mutation status, as well as other commonly implicated “actionable” or “druggable” oncogenes and tumour suppressor genes and correlated these with clinic-pathologic and outcome data.

## Methods

### Patients and tissue collection

Patients were identified from an institutional database of consecutive patients treated at the National Cancer Centre Singapore (NCCS) between January 1998 and March 2009. Included patients were confirmed to have a histological diagnosis of squamous cell carcinoma involving the anterior tongue, with complete clinico-pathologic data and fresh, frozen tumor samples available. Treatment decisions were made in weekly multi-disciplinary meetings and recorded prospectively. Only patients with no prior treatment for their cancers were included in this study and all patients were treated with upfront surgery followed by adjuvant therapy if applicable. Fresh tumor samples for sixty-six patients were retrieved from Singhealth Tissue Repository, with standardized written consent for use of clinical material (with covers tumor tissue, blood or other clinical specimens) and clinic-pathologic data for research. Both this study and the tissue collection/consent protocol have been approved by the Singhealth Centralized Institutional Review Board.

### Tissue preparation and DNA extraction

For all samples, tumor content was first determined by microscopic examination of hemotoxylin and Eosin (H&E) stained sections of the tissue by a board-certified pathology (TKL). DNA was only extracted from specimens determined to have > 50% tumor content. DNA extraction was performed using a 3 mm × 3 mm section of fresh-frozen tissue using the Qiamp DNA extraction kit (Qiagen, Valencia, CA) according to the manufacturer’s instructions. However, the sample DNA was eluted in molecular grade water instead of TE Buffer. The DNA concentration and purity were quantified and assessed using the Nanodrop 2000 spectrophotometer (Thermo Scientific, Wilmington, DE). All samples yielded excellent DNA quality with A260/A280 ratio greater than 1.60.

### Somatic mutation profiling using Sequenom LungCarta v1.0

Somatic mutation profiling was accomplished by using the Sequenom LungCarta v1.0 panel (http://www.sequenom.com) (Sequenom, San Diego, CA). The panel interrogated 214 somatic mutations across 26 oncogenes and tumor suppressors using the MassARRAY 4 System using multiplex PCR (Sequenom, San Diego, CA). Targeted mutation profiling was performed using the Sequenom Massarray 4 platform (Sequenom, San Diego, CA). Samples were evaluated for 214 mutations in a 24-multiplex PCR format using the LungCarta panel and analyzed on the matrix-assisted laser desorption ionization-time of flight mass spectrometry (MALDI-TOF) Sequenom platform. LungCarta panel provides evaluation of 214 somatic mutations in 26 oncogenes and tumour suppressor genes acting in key pathways in lung cancer (Table 1). Data extraction was performed using Sequenom MassArray Typer Analyzer software. Mutations were determined using a minimum 10% threshold of the mutant allele peak.

**Table 1:** **LungCarta 1.0- lung panel gene targets**

Gene	Mutation	Gene	Mutation
AKT1	E17K	MET	N375S, 982_1028del47
ALK	C1156Y, L1196M	NOTCH1	H2276fs*79, D1643H, R2328W, T1997M, V1672I, V2444fs*35
BRAF	D594G/M, G469S/E/A/V, L597Q/V, V600E/K/M	NRAS	Q61E/K/H/L/R/P
DDR2	C580Y, D125Y, G253C, G505S, G774E/V, I120M, I638F, L239R, L63V, T765P	NRF2	D29H, D77N/A, E79Q/K/G, G31A, G81D, R34Q,
EGFR	R108K, T263P, A289V, G598V, E709K/H, E709A/G/V, G719S/C/A/D, G719S/C/A/D, M766_A767insAI, D761Y/N, S768I, R776C/M, V769_D770insASV, V769_D770insCV, D770_N771 > AGG/V769_D770insASV/V769_D770insASV, D770_N771insG, N771_P772 > SVDNR, P772_H773insV, H773 > NPY, H773_V774insNPH/PH/H, V774_C775insHV, T790M, L858R/M, L861Q, E746_T751del, E746_A750del, E746_T751del, E746_T751del, S752D, L747_E749del, L747_T750del, L747_S752del, L747_T751del, L747_S752del, P753S, A750P, T751A, T751P, T751I, S752I/F, S752_I759del, L747_Q ins, E746_T751del, I ins (combined), E746_A750del, T751A (combined), L747_E749del, A750P (combined), L747_T750del, P ins (combined), L747_S752del, Q ins (combined), T854A	NTRK1	Q80*, R119H, S326R
EPHA3	A435S, D446Y, S449F, D806N, G187R, G518L, K761N, G766E, M269I, N379K, N85S, S229Y, T166N, T37K, T393K, W250R	NTRK2	Q666R, C45F, G261R, L138F, L670M, L755L
EPHA5	D493Y, G582E, M1034I, N1032S, R1007Q, S566Y, S810I, T856I	NTRK3	I769N, L152I, L248M, L270M, L336Q, S184C, T283K, V307L, R271F
ERBB2	M774_A775insAYVM, A775_G776insAYVM	PIK3CA	E542Q/K, E545Q/K. H1047Y/R/L
FGFR4	P672T, H192fs*19	PTCH1	R1308G, R682L, S1326fs*46
JAK2	L609S, P503L, R1122P, Y931C	PTEN	R233*
KRAS	G12S/V/F/R/A/C/D, G13C/S/A/V/DQ61L/R/P/H/E/K	PTPN11	E76V
MAP2K1	D67N, K57N, Q56P	PTPRD	D1162N, D154Y, I44I, L1036Q, P1809R, R1536L, R584S, S1703R, T337A, V483E
STK11	A347fs*13, A43_L50del6, D327fs*10, E120*, E165*, E223*, E70*, E70fs*26, F354L, G163C, G188fs*99, G196V, G56fs*4, G56W, G91L, H174R, I26fs*25, K191*, K78E, L285Q, L50_D53del4, M51fs*14, P179L, Q123R, Q137*, Q159*, Q170*, Q220*, Q37L, R426W, R86G, V197fs*69, V236fs*30, Y272Y	TP53	G245C/S, G245D/V, R158C/G/L/P, R175L/H, R248L/Q/R/W, R249S/W/M, R273C/H/L/P, R282G/W, V157F, Y163C,R175L/H Y220C

*Frameshift or truncating mutation.

An ion exchange resin (CLEAN Resin, Sequenom, San Diego, CA) was used to remove salt adducts. 41 μl of water was added to give a final volume of 50 ul after which the resin was added into the wells. The plates were incubated with the resin for 20 to 30 minutes. The samples were transferred onto the SpectroChip-II (96-well to 96-chip configuration) using the MassARRAY Nanodispener RS1000 (Sequenom, San Diego, CA). The dispense speed was at 70 mm/sec but was adjusted accordingly to ensure a consistent spotting volume range of 8 to 10 nL with a standard deviation of less than 3.5. The samples were resolved using the Matrix-Assisted Laser Desorption/Ionization Time-of-Flight (MALDI-TOF) mass spectrometer (Sequenom, San Diego, CA).

### Data analysis

Mutational analysis was accomplished using the Sequenom Typer Analyzer 4.0 software (Sequenom, San Diego, CA). The mutation detection threshold was set at 10%. The system provided a mutation list that showed the mutations that were picked up by the mass spectrometer. The mutations were sorted according to 3 different confidence levels (High, Medium and Low) based on peak height, morphology, statistical Z-score and allele frequencies [40, 41]. For all medium and high confidence calls, the electrophorograms were manually checked. For difficult cases, a second opinion was required. Only cases regarded as true are included in this list, and all the true cases in this situation were called with high confidence (see below). These experiments and analysis were performed in parallel with a series of lung cancer primary tissue and cell lines which served as positive controls for a range of mutations [40].

### Statistical analysis

Statistical analyses were performed using SPSS software. Student’s t test was used to compare group means while chi-square test and Fisher’s exact test were used to analyze other factors. Mean overall survival (OS) and disease-free survival (LRFS) were calculated. Kaplan-Meier plots were plotted and log-rank test was performed to compare the plots. In all statistical analyses, a p value of less than 0.05 was considered statistically significant.

## Results

Details of patient characteristics are shown in Table 2. In this cohort, 46 (69.7%) were male and 37 (56.1%) were ever-smokers. The median age at diagnosis was 63 years (range: 22-89 years). The majority of patients had locally advanced disease (T3/T4) (n = 40/60.6%). All patients in this cohort underwent surgery as the primary modality of treatment, and 36 (54.5%) received adjuvant radiation or chemo-radiation therapy. Patients were followed up for a median of 18 months (Range: 3 - 60 months). During this time period, there were 28 (42.4%) recurrences and 15 (22.7%) deaths. These were all cancers of the anterior tongue, and there was no evidence of involvement of human papillomavirus (HPV) in any of the tumors (manuscript in preparation- Iyer NG, Tan DSW).

**Table 2:** **Clinico-pathologic characteristics of patients with tongue squamous cell carcinoma in this study (n = 66)**

Characteristics	Number (%)
**Sex:**	
Male	46 (69.7%)
Female	20 (30.3%)
**Smoker:**	
Never	25 (37.9%)
Ever	37 (56.1%)
Unknown	4 (6.1%)
**Clinical T-classification**	
T1/T2	21 (31.8%)
T3/T4	40 (60.6%)
Tx	5 (7.6%)
**Clinical N-classification**	
N0	26 (39.4%)
N+	39 (59.1%)
Unknown	1 (1.5%)
**Adjuvant radiation or chemoradiation therapy:**	
Radiotherapy only	**30 (45.5%)**
Chemoradiotherapy	**6 (9.1%)**
N	20 (30.3%)
Unknown	10 (15.2%)
**Extra-capsular spread**	
Y	15 (22.7%)
N	16 (24.2%)
Not applicable	24 (36.4%)
Unknown	11 (16.7%)
**Lymphovascular and/or perineural invasion**	
Y	31 (47.0%)
N	20 (30.3%)
Unknown	15 (22.7%)
**Recurrence**	
Y	28 (42.4%)
N	29 (43.9%)
Unknown	14 (13.6%)

**Figure 1:**
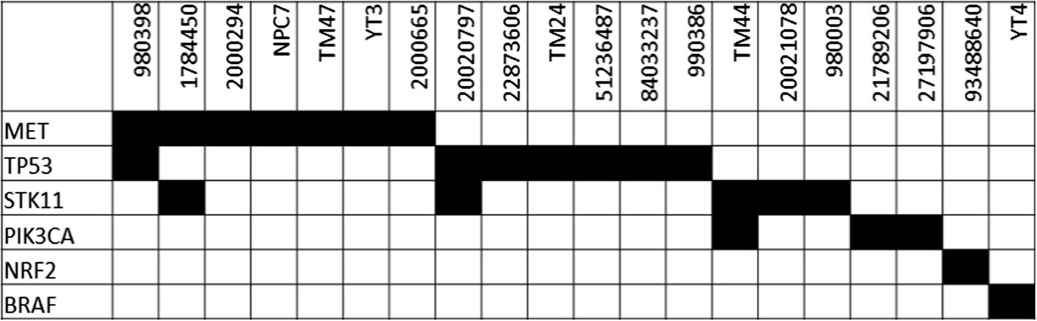
**Co-mutation map of samples identified to have at least one mutation in MET, TP53, STK11, PIK3CA, NRF2 and BRAF.**

Analyses of the Sequenom data showed that in total there were 42 high, 60 medium and 353 low confidence calls. Manual verification confirmed that of these 24 alterations were deemed to be true an included in further analysis, and all of these were derived from high confidence calls. The 24 alterations were identified in 20 (30.3%) tumors using the LungCarta 1.0 panel (Table 2). These were distributed across six genes: TP53, STK11, MET, PIK3CA, BRAF and NRF2 and the distribution of mutations across samples are indicated in Figure 1. There were thirteen unique alterations identified, with 16 tumors having a single variations and four tumors having two separate variations (Table 3). The commonest alterations seen were MET (10.6%; n = 7), TP53 (10.6%; n = 7), STK11 (9.1%; n = 6). Three PIK3CA mutations were “activating” (one with E542K and two with H1047N mutations). One tumor harbored a BRAF (D594G) mutation and another with a mutation in NRF2 (G31A), both of which are novel in TSCC, and the mutations have been validated through next-generation target re-sequencing of the same samples (manuscript in preparation). Tumor cells were propagated in culture as previously described [42]. In samples identified to have mutations, four (out of six attempted) have been successfully propagated in culture as cell lines: NPC7, TM24, TM44 and TM47 and profiling the cell lines confirmed that these harbored the same genetic alterations as the primary tumor they were derived from (Table 3).

**Table 3:** **Specific mutations and clinic-pathologic features samples where mutations were detected (n = 20)**

Sample	Gene	Mutation	TNM	Overall stage	Age/sex	Smoking status	Adjuvant treatment	Outcome	Time to recurrence (Months)
**Tumors with a single mutation**									
2000294	MET	N375S	TxN2bM1	4	53/M	Ever	Yes	Recurrence	7
20021078	STK11	F354L	T4aN1M0	4	79/M	Ever	Yes	No Recurrence	N.A.
21789206	PIK3CA	E542K	T4aN0M0	4	54/M	Ever	Yes	Recurrence	33
22873606	TP53	R175H	T4aN0M0	4	75/M	Ever	No	Unknown	N.A.
27197906	PIK3CA	H1047R	T4aN0M0	4	56/M	Ever	Yes	Unknown	N.A.
NPC7*	MET	N375S	T4N2bM0	4	22/F	Ever	Yes	Recurrence	6
TM24*	TP53	R282W	T2N2bM0	4	60/M	Ever	Yes	Recurrence	15
TM47*	MET	N375S	T4aN2cM0	4	76/M	Ever	Yes	Recurrence	6
93488640	NRF2	G31A	T2N1M0	3	61/M	Never	Yes	Recurrence	31
51236487	TP53	R273P	T2N0M0	2	60/M	Never	Yes	Unknown	N.A.
84033237	TP53	R273C	T4aN2bM0	4	56/M	Never	No	Recurrence	24
980003	STK11	F354L	T2N0M0	2	39/M	Never	No	Unknown	N.A.
YT3**	MET	N375S	T3N0M0	3	52/F	Never	No	Unknown	N.A.
YT4	BRAF	D594G	T1N0M0	1	42/F	Never	No	No Recurrence	N.A.
990386	TP53	R175H	T4aN0M0	4	63/M	Unknown	Unknown	No Recurrence	N.A.
2000665	MET	N375S	T4aN2bM0	4	53/F	Unknown	Yes	No Recurrence	N.A.
**Tumors with two separate mutations**									
980398	MET	N375S	T2N1M0	3	61/M	Never	No	No Recurrence	N.A.
	TP53	G245S							
1784450	MET	N375S	T4aN2cM0	4	66/M	Ever	No	Recurrence	5
	STK11	F354L							
20020797	STK11	F354L	T4aN2cM0	4	69/M	Ever	Yes	No Recurrence	N.A.
	TP53	Y163C							
TM44***	PIK3CA	H1047L	T2N2bM0	4	62/M	Ever	Yes	No Recurrence	N.A.
	STK11	F354L							

*Successfully propagated as cell line.

**Refused conventional treatment and lost to follow-up.

***Successfully propagated as cell line, patient had a 30-day mortality after primary surgery.

**Figure 2:**
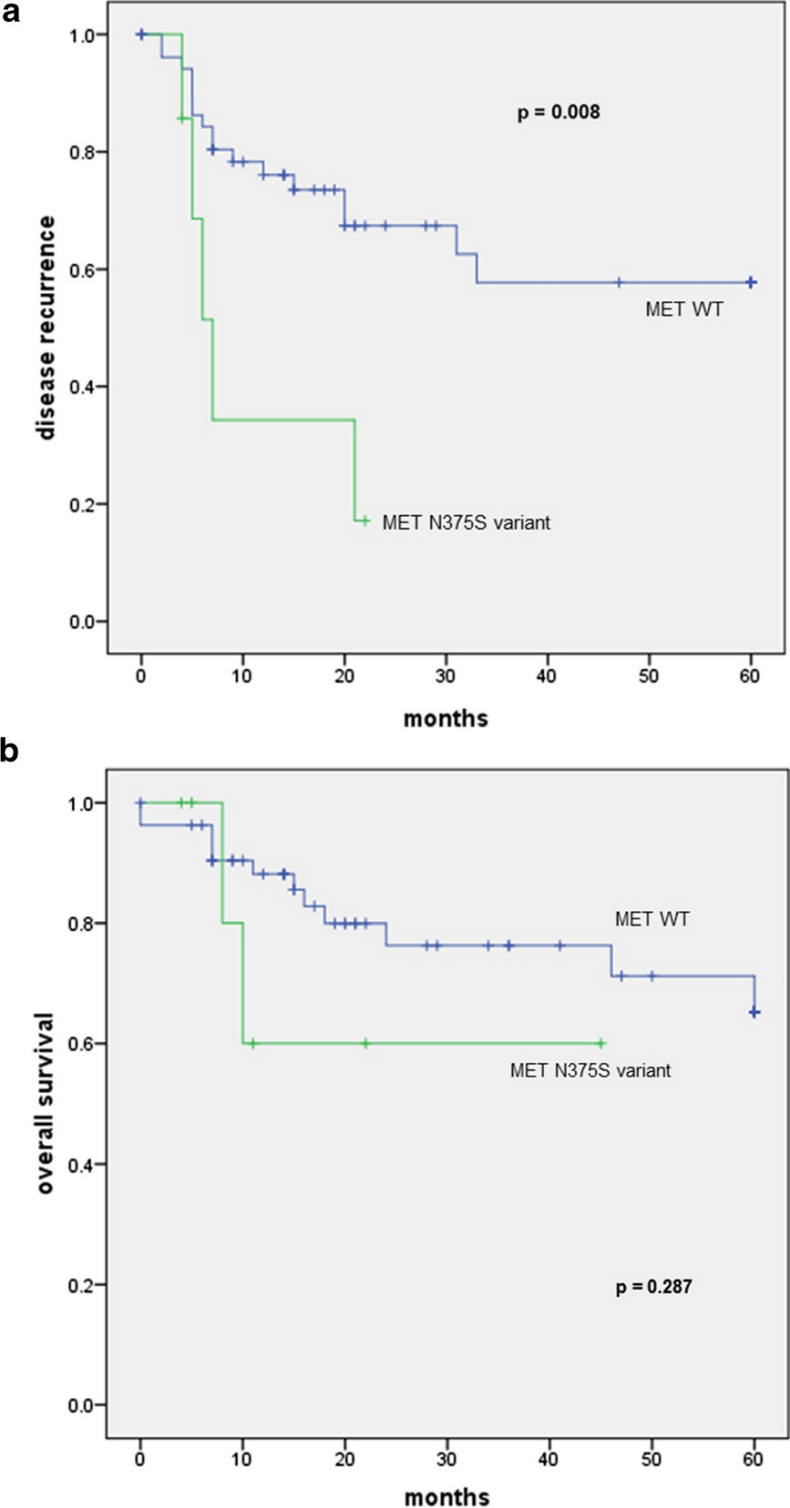
**Kaplan-Meier plots showing (a) Locoregional recurrence-free and (b) overall survival in patients with and without the MET N375S variant in the entire cohort.** P-value is computed based on the log rank test. Locoregional recurrence rates are significantly higher in patients with the MET 375S variant compared to those without.

There was no significant association between smoking history and the presence of any mutation detected by the LungCarta panel (p = 0.967), or specific alterations in MET (p = 0.806), p53 (p = 0.520) and STK11 (p = 0.105) (Table 3). There was also no correlation between patient age and mutation status (p = 0.360). With regards to outcome analyses, there were no significant correlations between the presence of any mutation and overall survival (OS) or loco-regional recurrence-free survival (LRFS). Median OS was 78 and 106 months (p = 0.711), while median LRFS was 56 and 93 months (p = 0.670) for patients with and without any mutations detected by this assay respectively. Similarly, there was no correlation between the presence of p53 or STK11 mutations and outcome. Interestingly, however, the presence of the MET N375S variant was associated with poorer loco-regional recurrence rates compared to patients who did not harbor this mutation: median LRFS of 11 versus 90 months respectively (p = 0.008) (Figure 2a). A trend to poorer survival was also seen when analyzing overall survival, where the median OS was 31 months in patients with MET mutation compared to 103 months in patients without although this was not statistically significant (p = 0.287) (Figure 2b).

## Discussion and conclusions

Oral squamous cell carcinoma remains a devastating disease with few treatment options in the metastatic setting. In this study, we used a panel designed for lung cancer to identify mutations in oral tongue cancer. Of the mutations tested, we only identified alterations in six genes in only 20 samples - TP53, STK11, MET, PIK3CA, BRAF and NRF2. Importantly, EGFR and KRAS mutations were not found in Asian tongue cancers, and this represents one of the largest series to date comprehensively profiled for these alterations. In contrast, other Asian cohorts, such as a large series from Korea, identified up to 14% of EGFR mutations in oral tongue SCC (n = 70), suggesting that population differences likely exist [36]. In addition, we did not observe any relationship between age, tobacco use and the prevalence of the mutations tested.

p53 mutations were the most common abnormality identified in 10.6% (7/66) of our patient cohort. While this low incidence can be explained by the fact that LungCarta only screens for twelve commonest hotspot mutations in the DNA binding domain, it may also reflect the high proportion of never smokers in our cohort. A recent study in Asian head and neck squamous cell carcinoma found that p53 mutations occurred in approximately 30% of HNSCC, in contrast to 60-80% in patients with risk factors such as smoking [43]. We did not find any significant relationship between p53 mutations and outcome, although this is limited by the small sample size.

The other common alteration identified in this panel is a specific MET variant (MET N357S), identified in 10.6% (7/66) of this cohort [44, 45]. The therapeutic role for inhibiting the MET pathway has yet to be validated, and several trials evaluating targeting MET are ongoing [46–48]. While this is likely a germline variant more commonly present in Asians (13%), it appears to confer resistance to MET inhibition by inhibiting ligand binding, suggesting that these patients would not benefit from currently available MET inhibitors [49]. Consistent with its role in cancer development, we found that patients with the variant had a poorer outcome than their wild type counterparts, with median loco-regional recurrence-free survival of 11 months compared to 90 months in patients without wild type MET (p = 0.008). Notwithstanding, these results warrant further investigation and should be validated in a larger series, to determine the prognostic value of MET alterations in HNSCC.

Other significant actionable alterations include activating mutations in PIK3CA (seen in 3 patients in our cohort) and STK11 (seen in six patients). These mutations have been described in HNSCC at similar frequencies [26, 38, 50] and are important considerations when planning future targeted therapy trials in TSCC. Two further mutations identified in this study are novel for TSCC. First, the BRAF mutation (D594G) seen here in one patient has not been previously reported in TSCC, and indeed other BRAF mutations are also exceedingly rare in HNSCC (<2%) [51]. The role of BRAF inhibitors targeting non-BRAF V600E cancers remains to be elucidated, although promising activity has been reported [52, 53]. NRF2 mutation was also identified in this cohort consistent with the The Cancer Genome Atlas (TCGA) database where mutations in NFEL2 (which is the gene for NRF2) occur in 6% of HNSCC [24]. NRF2 is a transcription factor that regulates cellular response to oxidative stress by inducing the expression of cytoprotective proteins. It is often overexpressed in human cancers and somatic mutations have been detected in lung cancer [54–56].

The paucity of mutations found using the LungCarta panel suggests that profiling of head and neck cancers would require a more customised approach and a better design of a panel focused on head and neck cancers, and this is in our future pipeline. It is however imperative to ensure high-tumor percentage in tissues examine to ensure that a lack of mutations reflects a true negative result rather than false negatives due to a high proportion of normal cells. If necessary this can be mitigated by macro- or micro-dissection to enrich for tumor cells. Large scale next-generation sequencing efforts from organizations such as TCGA and ICGC would be invaluable in delineating the molecular basis of OSCC. The challenge for the post-genomic/post-discovery era requires implementing pragmatic strategies to identify alterations and pathways that are therapeutically tractable. In this regard, mass spectrometry based mutation profiling is a robust technique that can be applied on frozen or formalin-fixed embedded tissue, and is particularly well-suited for multiplexed screening of hotspot alterations in large patient cohorts [40].

Other issues in this study include a lack of normal patient DNA to confirm that these finding are truly somatic. However, with increasing data available form large scale sequencing efforts and data available in the Cosmic database, panels can be specifically designed to only examine alterations know to be somatic. Notwithstanding it is also important to consider germline variants that can have effects on outcome and therapeutic decisions. The other deficiency with this approach is that it is focused on hotspot mutations which are predictable and limited, and hence not effective in detecting inactivating mutations which tend to be more widely distributed. In this regard, a capture technique (such as those offered by Ion Torrent or Sureselect systems) may be more inclusive. However, unlike Sequenom, the latter often require validation using Sanger sequencing to confirm findings. This study relied on the availability of sufficient tumor specimen to perform these analyses; hence there were fewer small, early T-stage tumors in our cohort than normally seen in the clinic, despite being a consecutive series.

In conclusion, we confirm that common activating EGFR mutations and KRAS mutations are not present in a large cohort of Asian tongue cancers. Molecular profiling using Sequenom MA4 is a robust, reproducible technique in determining clinically relevant mutations e.g. PIK3CA [39, 40]. Patient derived cell lines that recapitulate specific genetic changes in the original tumour are important discovery tools that will facilitate further understanding of cell context-specific factors of genetic alterations. Future design of a specific HNSCC panel should take into consideration important actionable mutations identified through the ongoing large scale sequencing efforts.

## Abbreviations

TSCC: Tongue squamous cell carcinomaOSCC, Oral squamous cell carcinoma
TKI: Tyrosine kinase inhibitor
OS: Overall survival
LRFS: Loco-regional recurrence free survival
ICGC: International Cancer Genome Consortium
NSCLC: Non-small cell lung cancer
HNSCC: Head and neck squamous cell carcinoma
HPV: Human papillomavirus
TCGA: The Cancer Genome Atlas.
